# Supplementary material for: Association of sleep duration in middle and old age with incidence of dementia
Source: Nat Commun. 2021 Apr 20;12:2289. doi: 10.1038/s41467-021-22354-2 (PMC8058039; doi:10.1038/s41467-021-22354-2)
Supplement: Supplementary file 1 — Supplementary Information [file 41467_2021_22354_MOESM1_ESM.pdf]

Association of sleep duration in middle and old age with incidence of dementia

Séverine Sabia, Aurore Fayosse, Julien Dumurgier, Vincent T. van Hees, Claire Paquet, Andrew Sommerlad, Mika Kivimäki, Aline Dugravot, Archana Singh-Manoux

Supplementary material content

Supplementary Figure 1. Nelson-Aalen cumulative hazards of dementia by sleep duration at age 50, 60, and 70

Supplementary Table 1. Sample characteristics at age 60 and 70

Supplementary Table 2. Sample characteristics of the accelerometer substudy in 2012-2013

Supplementary Table 3. Sensitivity analysis: use of APOEε4 as a covariate in the analysis

Supplementary Table 4. Sensitivity analysis: association of sleep duration with a proxy for Alzheimer's disease dementia

Supplementary Figure 1. Nelson-Aalen cumulative hazards of dementia by sleep duration at age 50, 60, and 70

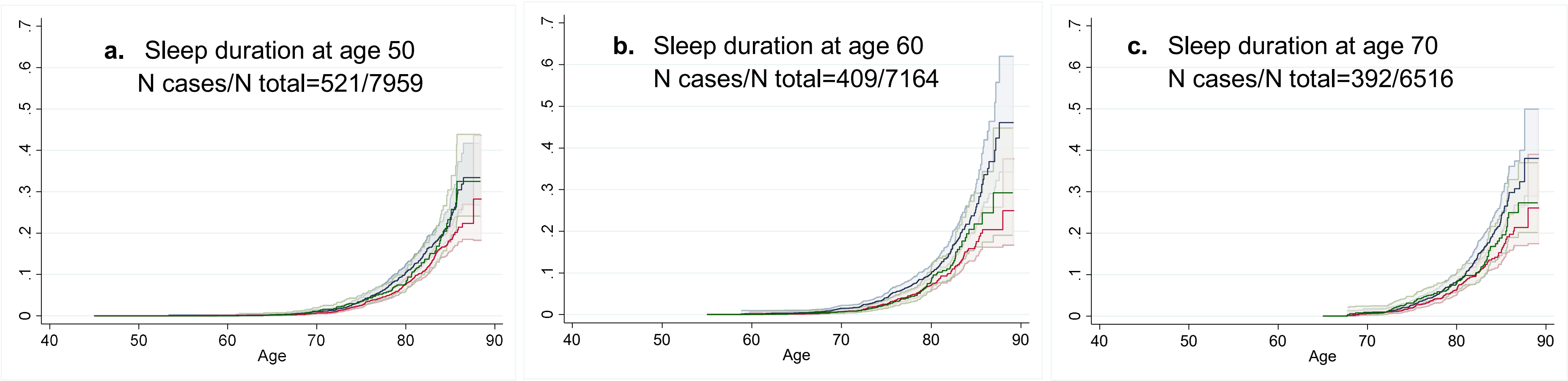

a represents Nelson-Aalen cumulative hazards of dementia and the corresponding 95% confidence interval for each sleep duration group defined at age 50 (dark blue, sleep duration ≤6 hours (N cases/N total=211/3149); red, sleep duration = 7 hours (N cases/N total=219/3624); green, sleep duration ≥8 hours (N cases/N total=91/1186))

b represents Nelson-Aalen cumulative hazards of dementia and the corresponding 95% confidence interval for each sleep duration group defined at age 60 (dark blue, sleep duration ≤6 hours (N cases/N total=192/2759); red, sleep duration = 7 hours (N cases/N total=142/2988); green, sleep duration ≥8 hours (N cases/N total=75/1417))

c represents Nelson-Aalen cumulative hazards of dementia and the corresponding 95% confidence interval for each sleep duration group defined at age 70 (dark blue, sleep duration ≤6 hours (N cases/N total=171/2429); red, sleep duration = 7 hours (N cases/N total=131/2578); green, sleep duration ≥8 hours (N cases/N total=90/1509))

Supplementary Table 1. Sample characteristics at age 60 and 70

	Sleep duration at age 60				Sleep duration at age 70			
	Short: ≤6 hours	Normal: 7 hours	Long: ≥8 hours	P	Short: ≤6 hours	Normal: 7 hours	Long: ≥8 hours	P
N	2759	2988	1417		2429	2578	1509	
Women	911 (33.0)	829 (27.7)	412 (29.1)	<0.001	878 (36.2)	653 (25.3)	394 (26.1)	<0.001
Ethnicity, Non-white	283 (10.3)	195 (6.5)	104 (7.3)	<0.001	254 (10.5)	165 (6.4)	117 (7.8)	<0.001
Less than secondary school diploma	1261 (45.7)	1282 (42.9)	605 (42.7)	0.06	1175 (48.4)	1125 (43.6)	658 (43.6)	0.001
Married/cohabiting	1980 (71.8)	2345 (78.5)	1129 (79.7)	<0.001	1642 (67.6)	1995 (77.4)	1194 (79.1)	<0.001
Moderate alcohol drinking	1403 (50.9)	1583 (53.0)	740 (52.2)	0.27	1259 (51.8)	1468 (56.9)	782 (51.8)	<0.001
Current smokers	272 (9.9)	261 (8.7)	130 (9.2)	0.34	131 (5.4)	119 (4.6)	80 (5.3)	0.41
Moderate-to-vigorous physical activity (hours), M(SD)	3.4 (3.4)	3.8 (3.5)	4.0 (3.7)	<0.001	3.2 (3.3)	4.0 (3.5)	3.9 (3.9)	<0.001
Daily fruit and vegetable consumption	2022 (73.3)	2283 (76.4)	1105 (78.0)	0.001	1813 (74.6)	2102 (81.5)	1220 (80.9)	<0.001
BMI≥30 kg/m²	572 (20.7)	472 (15.8)	213 (15.0)	<0.001	549 (22.6)	441 (17.1)	297 (19.7)	<0.001
Diabetes	249 (9.1)	184 (6.2)	98 (6.9)	<0.001	362 (14.9)	310 (12.0)	196 (13.0)	0.01
Hypertension	1062 (38.5)	1107 (37.1)	526 (37.1)	0.48	1343 (55.3)	1361 (52.8)	813 (53.9)	0.21
Cardiovascular disease	267 (9.7)	231 (7.7)	113 (8.0)	0.02	445 (18.3)	346 (13.4)	250 (16.6)	<0.001
GHQ depression	415 (15.0)	252 (8.4)	110 (7.8)	<0.001	338 (13.9)	158 (6.1)	106 (7.0)	<0.001
CNS medications	160 (5.8)	120 (4.0)	84 (5.9)	0.002	167 (6.9)	123 (4.8)	121 (8.0)	<0.001
Mental disorders before age 65	239 (8.7)	209 (7.0)	129 (9.1)	0.02	192 (7.9)	160 (6.2)	118 (7.8)	0.04

Abbreviations: BMI, body mass index; CNS, central nervous system; GHQ, general health questionnaire; M, mean; SD, standard deviation.

Values are No. (%) unless stated otherwise. Two-sided Ps for heterogeneity were estimated using χ^2 test for categorical variables, and ANOVA for continuous variables.

Supplementary Table 2. Sample characteristics of the accelerometer substudy in 2012-2013

	Sleep duration in 2012-2013			
	Tertile 1<br>1 h 16 min<br>to 6 h 13 min	Tertile 2<br>6 h 14 min<br>to 7 h 0 min	Tertile 3<br>7 h 1 min to<br>10 h 6 min	P
N	1296	1296	1296	
Age, M(SD)	69.4 (5.7)	69.3 (5.7)	69.3 (5.7)	0.94
Women	279 (21.5)	362 (27.9)	369 (28.5)	<0.001
Ethnicity, Non-white	126 (9.7)	75 (5.8)	81 (6.3)	<0.001
Less than secondary school diploma	524 (40.4)	544 (42.0)	537 (41.4)	0.72
Married/cohabiting	924 (71.3)	973 (75.1)	1014 (78.2)	<0.001
Moderate alcohol drinking	719 (55.5)	774 (59.7)	716 (55.3)	0.04
Current smokers	50 (3.9)	42 (3.2)	30 (2.3)	0.08
Moderate-to-vigorous physical activity (hours), M(SD)	3.4 (3.6)	3.6 (3.8)	3.6 (3.4)	0.14
Daily fruit and vegetable consumption	987 (76.2)	1060 (81.8)	1042 (80.4)	0.001
BMI≥30 kg/m²	284 (21.9)	211 (16.3)	208 (16.1)	<0.001
Diabetes	189 (14.6)	150 (11.6)	159 (12.3)	0.06
Hypertension	688 (53.1)	680 (52.5)	651 (50.2)	0.31
Cardiovascular disease	222 (17.1)	203 (15.7)	213 (16.4)	0.60
GHQ depression	114 (8.8)	111 (8.6)	97 (7.5)	0.43
CNS medications	76 (5.9)	65 (5.0)	100 (7.7)	0.01
Mental disorders before age 65	95 (7.3)	95 (7.3)	122 (9.4)	0.08

Abbreviations: BMI, body mass index; CNS, central nervous system; GHQ, general health questionnaire; M, mean; SD, standard deviation.

Values are No. (%) unless stated otherwise. Two-sided Ps for heterogeneity were estimated using χ^2 test for categorical variables, and ANOVA for continuous variables.

Supplementary Table 3. Sensitivity analysis: use of APOEε4 as a covariate in the analysis

		Model not adjusted for APOEε4		Model adjusted for APOEε4	
	N cases/ N total	HR[a] (95% CI)	P-value[b]	HR[c] (95% CI)	P-value[b]
Sleep duration at age 50					
Short: ≤ 6 hours	113/1961	1.27 (0.98-1.65)	0.07	1.25 (0.96-1.62)	0.10
Normal: 7 hours	121/2322	1 (ref)		1 (ref)	
Long: ≥ 8 hours	41/693	1.24 (0.87-1.77)	0.24	1.23 (0.86-1.76)	0.25
Sleep duration at age 60					
Short: ≤ 6 hours	119/1926	1.42 (1.08-1.87)	0.01	1.42 (1.08-1.86)	0.01
Normal: 7 hours	95/2260	1 (ref)		1 (ref)	
Long: ≥ 8 hours	52/1039	1.19 (0.84-1.66)	0.33	1.21 (0.86-1.70)	0.27
Sleep duration at age 70					
Short: ≤ 6 hours	114/1768	1.14 (0.87-1.49)	0.35	1.12 (0.85-1.47)	0.41
Normal: 7 hours	103/1990	1 (ref)		1 (ref)	
Long: ≥ 8 hours	56/1135	0.90 (0.65-1.25)	0.54	0.93 (0.67-1.29)	0.65
Trajectories of sleep duration between age 50 and 70					
Persistent short	59/947	1.14 (0.82-1.59)	0.42	1.12 (0.81-1.56)	0.49
Persistent normal	103/1894	1 (ref)		1 (ref)	
Persistent long	19/334	0.95 (0.58-1.57)	0.85	0.95 (0.58-1.57)	0.85
Change from short to normal	49/850	1.29 (0.92-1.83)	0.14	1.22 (0.86-1.72)	0.26
Change from normal to long	28/721	0.80 (0.52-1.22)	0.29	0.82 (0.54-1.25)	0.36
Change from normal to short	21/320	1.00 (0.62-1.60)	0.99	0.94 (0.58-1.51)	0.79
Accelerometer-assessed sleep duration					
Tertile 1: 1 h 16 min to 6 h 13 min	42/1040	1.66 (1.00-2.75)	0.05	1.62 (0.97-2.69)	0.07
Tertile 2: 6 h 14 min to 7 h 0 min	25/1094	1 (ref)		1 (ref)	
Tertile 3: 7 h 1 min to 10 h 6 min	19/1093	0.67 (0.36-1.23)	0.20	0.69 (0.37-1.27)	0.23

Abbreviations: CI, confidence intervals; HR, hazard ratio; IPW, inverse probability weighting.
[a] HR estimated from a Cox regression adjusted for age (time-scale), sex, ethnicity, education, and marital status, alcohol consumption, physical activity, smoking status, and fruit and vegetable consumption, BMI, hypertension, diabetes, CVD, GHQ depression, and CNS medications.
[b] Two-sided P-value for HR in comparison with the reference (ref) category, without adjustment for multiple comparisons.
[c] Model additionally adjusted for APOE ε4 (1 or 2, versus 0) allele.

Supplementary Table 4. Sensitivity analysis: association of sleep duration with a proxy for Alzheimer's disease dementia

	All cause dementia			Alzheimer's disease type of dementia[a]		
	N cases/ N total	HR[b] (95% CI)	P-value[c]	N cases/ N total	HR[c] (95% CI)	P-value[c]
Sleep duration at age 50						
Short: ≤ 6 hours	211/3149	1.22 (1.01-1.48)	0.04	159/3149	1.13 (0.91-1.40)	0.27
Normal: 7 hours	219/3624	1 (ref)		178/3624	1 (ref)	
Long: ≥ 8 hours	91/1186	1.25 (0.98-1.60)	0.07	67/1186	1.13 (0.85-1.50)	0.39
Sleep duration at age 60						
Short: ≤ 6 hours	192/2759	1.37 (1.10-1.72)	0.005	147/2759	1.27 (0.99-1.63)	0.06
Normal: 7 hours	142/2988	1 (ref)		119/2988	1 (ref)	
Long: ≥ 8 hours	75/1417	1.15 (0.87-1.52)	0.34	58/1417	1.06 (0.77-1.45)	0.72
Sleep duration at age 70						
Short: ≤ 6 hours	171/2429	1.24 (0.98-1.57)	0.10	131/2429	1.22 (0.94-1.59)	0.14
Normal: 7 hours	131/2578	1 (ref)		105/2578	1 (ref)	
Long: ≥ 8 hours	90/1509	1.15 (0.88-1.51)	0.60	68/1509	1.11 (0.81-1.51)	0.52
Trajectories of sleep duration between age 50 and 70						
Persistent short	103/1358	1.30 (1.00-1.69)	0.048	78/1358	1.22 (0.91-1.64)	0.19
Persistent normal	141/2520	1 (ref)		116/2520	1 (ref)	
Persistent long	35/461	1.28 (0.88-1.85)	0.20	27/461	1.20 (0.79-1.83)	0.40
Change from short to normal	61/1086	1.20 (0.89-1.63)	0.23	46/1086	1.11 (0.78-1.56)	0.56
Change from normal to long	47/946	1.02 (0.73-1.42)	0.90	37/946	1.00 (0.69-1.45)	0.99
Change from normal to short	39/504	1.13 (0.79-1.62)	0.50	29/504	1.06 (0.70-1.61)	0.77
Accelerometer-assessed sleep duration						
Tertile 1: 1 h 16 min to 6 h 13 min	53/1296	1.63 (1.04-2.57)	0.03	43/1296	1.56 (0.95-2.55)	0.08
Tertile 2: 6 h 14 min to 7 h 0 min	31/1296	1 (ref)		27/1296	1 (ref)	
Tertile 3: 7 h 1 min to 10 h 6 min	27/1296	0.78 (0.46-1.32)	0.36	18/1296	0.61 (0.33-1.11)	0.33

Abbreviations: CI, confidence intervals; HR, hazard ratio; IPW, inverse probability weighting.
[a] Alzheimer's type of dementia definition is based on dementia cases without cardiovascular disease history (stroke or myocardial infarction).
[b] HR estimated from a Cox regression adjusted for age (time-scale), sex, ethnicity, education, and marital status, alcohol consumption, physical activity, smoking status, and fruit and vegetable consumption, BMI, hypertension, diabetes, CVD, GHQ depression, and CNS medications.
[c] Two-sided P-value for HR in comparison with the reference (ref) category, without adjustment for multiple comparisons.

Supplementary STROBE Statement—checklist of items that should be included in reports of observational studies

	Item No.	Recommendation	Page No.	Relevant text from manuscript
**Title and abstract**	1	(*a*) Indicate the study's design with a commonly used term in the title or the abstract	1	Association of sleep duration in middle and old age with incidence of dementia
		(*b*) Provide in the abstract an informative and balanced summary of what was done and what was found	2	See details on page 2
**Introduction**				
Background/rationale	2	Explain the scientific background and rationale for the investigation being reported	3	Two first paragraphs of page 3
Objectives	3	State specific objectives, including any prespecified hypotheses	3	Third paragraph of page 3
**Methods**				
Study design	4	Present key elements of study design early in the paper	11	Paragraph on study population
Setting	5	Describe the setting, locations, and relevant dates, including periods of recruitment, exposure, follow-up, and data collection	11 to 13	Paragraph on study population, sleep duration, and dementia
Participants	6	(*a*) *Cohort study*—Give the eligibility criteria, and the sources and methods of selection of participants. Describe methods of follow-up<br><br>*Case-control study*—Give the eligibility criteria, and the sources and methods of case ascertainment and control selection. Give the rationale for the choice of cases and controls<br><br>*Cross-sectional study*—Give the eligibility criteria, and the sources and methods of selection of participants	11	Paragraph on study population + flow chart of the study in Supplementary Figure 1
		(*b*) *Cohort study*—For matched studies, give matching criteria and number of exposed and unexposed<br><br>*Case-control study*—For matched studies, give matching criteria and the number of controls per case		Not appropriate
Variables	7	Clearly define all outcomes, exposures, predictors, potential confounders, and effect modifiers. Give diagnostic criteria, if applicable	11-14	Paragraphs on sleep duration, dementia, and covariates
Data sources/ measurement	8*	For each variable of interest, give sources of data and details of methods of assessment (measurement). Describe comparability of assessment methods if there is more than one group	11-14	Paragraphs on sleep duration, dementia, and covariates
Bias	9	Describe any efforts to address potential sources of bias	15	Use of accelerometer data to account for potential reporting bias

				and of inverse probability weighting to account for missing data
Study size	10	Explain how the study size was arrived at	4-5	First paragraph of the results section + flow chart in Supplementary Figure 1 + last paragraph on page 5
Quantitative variables	11	Explain how quantitative variables were handled in the analyses. If applicable, describe which groupings were chosen and why	11-12	Paragraph on sleep duration
Statistical methods	12	(*a*) Describe all statistical methods, including those used to control for confounding	14	First paragraph of page 14.
		(*b*) Describe any methods used to examine subgroups and interactions	4, 28	No evidence of interaction between sex and variables of interest, exclusion of history of mental disorders was also considered (table 4)
		(*c*) Explain how missing data were addressed	15	Missing data on the exposure variable (sleep duration) was accounted for by conducted a sensitivity analysis using inverse probability weighting (page 15, 3rd paragraph)
		(*d*) *Cohort study*—If applicable, explain how loss to follow-up was addressed<br>*Case-control study*—If applicable, explain how matching of cases and controls was addressed<br>*Cross-sectional study*—If applicable, describe analytical methods taking account of sampling strategy	11	There was no problem of loss of follow-up as dementia was ascertained using linkage to electronic health records for all participants.
		(*e*) Describe any sensitivity analyses	15	Third paragraph of page 15
**Results**				
Participants	13*	(a) Report numbers of individuals at each stage of study—eg numbers potentially eligible, examined for eligibility, confirmed eligible, included in the study, completing follow-up, and analysed	4	First paragraph of the results section, Supplementary Figure 1
		(b) Give reasons for non-participation at each stage	4	First paragraph of the results section, Supplementary Figure 1
		(c) Consider use of a flow diagram	4	Supplementary Figure 1

Descriptive data	14*	(a) Give characteristics of study participants (eg demographic, clinical, social) and information on exposures and potential confounders	4	Second paragraph of the results section
		(b) Indicate number of participants with missing data for each variable of interest	4	Supplementary Figure 1
		(c) *Cohort study*—Summarise follow-up time (eg, average and total amount)	5 - 6 + 26 - 29	Tables 2 to 5 footnotes as well as in the results sections
Outcome data	15*	*Cohort study*—Report numbers of outcome events or summary measures over time	5 - 6 + 26 - 29	Tables 2 to 5 footnotes as well as in the results sections
		Case-control study—Report numbers in each exposure category, or summary measures of exposure		
		Cross-sectional study—Report numbers of outcome events or summary measures		
Main results	16	(*a*) Give unadjusted estimates and, if applicable, confounder-adjusted estimates and their precision (eg, 95% confidence interval). Make clear which confounders were adjusted for and why they were included	26 - 29	Tables 2 to 5 : Several levels of adjustment are provided in the tables for HR as well as unadjusted incident rate.
		(*b*) Report category boundaries when continuous variables were categorized	29	Boundaries of tertiles of sleep duration assessed by accelerometer data are provided in table 5. Sleep duration in tables 1 to 4 was a categorical variable.
		(*c*) If relevant, consider translating estimates of relative risk into absolute risk for a meaningful time period		Tables 2, 3, and 5 include incidence rate as well as HRs
Other analyses	17	Report other analyses done—eg analyses of subgroups and interactions, and sensitivity analyses	6-7	Sensitivity analyses are described in the Sensitivity analysis paragraphs of the Results section
**Discussion**				
Key results	18	Summarise key results with reference to study objectives	7	First paragraph of the discussion section
Limitations	19	Discuss limitations of the study, taking into account sources of potential bias or imprecision. Discuss both direction and magnitude of any potential bias	10	See sections on strengths and limitations of the study

Interpretation	20	Give a cautious overall interpretation of results considering objectives, limitations, multiplicity of analyses, results from similar studies, and other relevant evidence	10-11	Last paragraph of the discussion section
Generalisability	21	Discuss the generalisability (external validity) of the study results	10	See 2nd paragraph of page 10
**Other information**				
Funding	22	Give the source of funding and the role of the funders for the present study and, if applicable, for the original study on which the present article is based	22	See page 22 for funding section

*Give information separately for cases and controls in case-control studies and, if applicable, for exposed and unexposed groups in cohort and cross-sectional studies.

Note: An Explanation and Elaboration article discusses each checklist item and gives methodological background and published examples of transparent reporting. The STROBE checklist is best used in conjunction with this article (freely available on the Web sites of PLoS Medicine at http://www.plosmedicine.org/, Annals of Internal Medicine at http://www.annals.org/, and Epidemiology at http://www.epidem.com/). Information on the STROBE Initiative is available at www.strobe-statement.org.
